# The paralogous R3 MYB proteins CAPRICE, TRIPTYCHON and ENHANCER OF TRY AND CPC1 play pleiotropic and partly non-redundant roles in the phosphate starvation response of *Arabidopsis* roots

**DOI:** 10.1093/jxb/erv259

**Published:** 2015-05-28

**Authors:** Chun-Ying Chen, Wolfgang Schmidt

**Affiliations:** ^1^Institute of Plant and Microbial Biology, Academia Sinica, Taipei, Taiwan; ^2^Molecular and Biological Agricultural Sciences Program, Taiwan International Graduate Program, Academia Sinica, Taipei, Taiwan, and National Chung-Hsing University, Taichung, Taiwan; ^3^Graduate Institute of Biotechnology, National Chung-Hsing University, Taichung, Taiwan; ^4^Biotechnology Center, National Chung-Hsing University, Taichung, Taiwan; ^5^Genome and Systems Biology Degree Program, College of Life Science, National Taiwan University, Taipei, Taiwan

**Keywords:** Gene regulation, genetic redundancy, phosphate starvation, RNA-seq, root hairs, transcriptional profiling.

## Abstract

Phosphate (Pi) deficiency alters root hair length and frequency as a means of increasing the absorptive surface area of roots. Three partly redundant single R3 MYB proteins, CAPRICE (CPC), ENHANCER OF TRY AND CPC1 (ETC1) and TRIPTYCHON (TRY), positively regulate the root hair cell fate by participating in a lateral inhibition mechanism. To identify putative targets and processes that are controlled by these three transcription factors (TFs), we conducted transcriptional profiling of roots from *Arabidopsis thaliana* wild-type plants, and *cpc*, *etc1* and *try* mutants grown under Pi-replete and Pi-deficient conditions using RNA-seq. The data show that in an intricate interplay between the three MYBs regulate several developmental, physiological and metabolic processes that are putatively located in different tissues. When grown on media with a low Pi concentration, all three TFs acquire additional functions that are related to the Pi starvation response, including transition metal transport, membrane lipid remodelling, and the acquisition, uptake and storage of Pi. Control of gene activity is partly mediated through the regulation of potential antisense transcripts. The current dataset extends the known functions of R3 MYB proteins, provides a suite of novel candidates with critical function in root hair development under both control and Pi-deficient conditions, and challenges the definition of genetic redundancy by demonstrating that environmental perturbations may confer specific functions to orthologous proteins that could have similar roles under control conditions.

## Highlight

RNA sequencing of mutants defective in the expression of three paralogous MYB transcription factors revealed pleiotropic roles and dynamic shifts in the function of the proteins upon exposure to phosphate starvation.

## Introduction

Deviations from intrinsic developmental patterns, dictated by environmental signals, confer phenotypic plasticity to plants and allow for an efficient acclimation to prevailing conditions. Root hairs, specialized epidermal cells that play critical roles in the absorption of water and nutrients, provide an excellent model for studying such changes. While genetically determined, the development of root epidermal cells is highly responsive to the environment, resulting in alterations in both the density and length of root hairs. This response is specifically triggered by suboptimal phytoavailability of mineral nutrients with low mobility such as phosphate (Pi), iron and manganese (Perry *et al.*, 2007). In *Arabidopsis*, root hairs develop from files of epidermal cells that are positioned over the clefts of two underlying cortical cells (H position), triggered by a yet unidentified positional signal. This ‘cortical bias’ is perceived by the leucine-rich receptor kinase SCRAMBLED (SCM) (Kwak *et al.*, 2005). SCM activity is regulated in turn by the zinc finger protein JACKDAW in a non-cell autonomous manner through a signal from the underlying cortex cell layer. This signal is presumably stronger in H-positioned cells than in epidermal cells adjacent to periclinal cortical cell walls (Hassan *et al.*, 2010). SCM represses the expression of the R2R3 MYB proteins *WEREWOLF* (*WER*) and *MYB23* in future hair cells (trichoblasts). In non-hair cells, WER forms a complex with the WD repeat protein TTG, the basic helix-loop-helix protein GLABRA3 (GL3), and its paralogue ENHANCER OF GLABRA3 (EGL3). This complex supports the expression of the homeodomain-leucine zipper protein GLABRA2 (GL2), which represses the root hair cell fate. Four R3 MYB proteins with putatively redundant function, CAPRICE (CPC), ENHANCER OF TRY AND CPC 1 (ETC1), ENHANCER OF TRY AND CPC3 (ETC3), and TRIPTYCHON (TRY), compete with WER for binding to the TTG-GL3/EGL3 complex in a lateral inhibition mechanism. In this mechanism, R3 MYB proteins migrate, possibly via plasmodesmata, from non-hair cells to hairs cells where they are trapped by EGL3 (Kang *et al.*, 2013). The movement of R3 MYBs from non-hair cells to hair cells and the reduced expression of *WER* and *MYB23* repress the non-hair pathway and force cells in the H-position into the hair fate.

CPC and TRY act redundantly in trichome and root hair patterning (Schellmann *et al.*, 2007), but TRY has a specific role in regulating the expression of *SCM* (Kwak *et al.*, 2005; Kwak and Schiefelbein, 2014), indicating specific, partly non-redundant functions of the R3 MYB proteins. Three other R3 MYBs, ETC2, TCL1 and TCL2, are not expressed in roots and play a role in trichome formation (Wang *et al.*, 2007; Kirik *et al.*, 2004*b*). ETC1, ETC2 and ETC3 possess similar biochemical properties and can substitute for CPC function by acting in a mechanistically similar manner (Kirik *et al.*, 2004*a*; Simon *et al.*, 2007; Tominaga *et al.*, 2008). In support of this proposition, the number of root hairs is significantly decreased in *etc3* mutants (Tominaga *et al.*, 2008; Wester *et al.*, 2009).

*GL3* and *EGL3* are preferentially expressed in root hair cells, but the proteins can migrate to non-hair cells to reinforce the cell fate decisions (Bernhardt *et al.*, 2005). In cells that develop into root hairs, the CPC/ETC1/TRY-GL3/EGL3-TGG complex represses the expression of *GL2*, and cells enter the hair cell fate (Wada *et al.*, 2002). GL2 negatively regulates the expression of the bHLH-type transcription factor (TF) ROOT HAIR DEFECTIVE6 (RHD6), which is essential for the assembly of the root hair initiation site (Masucci and Schiefelbein, 1994). Root hair initiation starts with the formation of a dome-shaped structure at the basal end of the trichoblast. ROOT HAIR DEFECTIVE6-LIKE4 (RSL4) is a direct transcriptional target of RHD6 and controls a suite of genes involved in the elongation of the hairs (Yi *et al.* 2010).

Pi-deficient plants form longer and more frequent root hairs than plants grown under Pi-replete conditions (Ma *et al.*, 2001; Müller and Schmidt, 2004). This increase in root hair number is primarily caused by a decrease of the longitudinal length of epidermal cells (Ma *et al.*, 2003; Savage *et al.*, 2013). In addition, in roots of Pi-deficient plants a small fraction of root hairs are formed in positions normally occupied by non-hair cells (Ma *et al.*, 2001; Müller and Schmidt, 2004; Savage *et al.*, 2013). The restricted elongation of root epidermal cells under conditions of Pi deficiency is caused by reduced strength of the positional signal that determines root hair patterning via SCM. This scenario was inferred from the observation that *scm* mutants develop short, trichoblast-like epidermal cells (Savage *et al.*, 2013). RSL4 controls the duration of root hair elongation and is thus critical for later phases of root hair development (Yi *et al.*, 2010; Wada *et al.*, 2015). Expression of *RSL4* is responsive to Pi availability. Increased activity of ETC1 upon Pi starvation has been proposed as a mechanism for conferring additional root hair cell fate assignment. Overexpression of *ETC1* causes the formation of excessive root hairs (Kirik *et al.* 2004*a*), supporting the assumption that ETC1 and CPC have similar functions. In *cpc etc1* double mutants the formation of extra root hairs in response to Pi deficiency was abolished (Savage *et al.*, 2013), and *ETC1* was identified as a putative target of the homeodomain protein ALFIN LIKE6 (AL6), a PHD finger-containing ‘histone reader’ that is critical for the elongation of Pi deficiency-induced root hairs (Chandrika *et al.*, 2013). AL6 binds to trimethylated lysine 4 on histone 3 (Lee *et al.*, 2009) and probably controls gene activity by recruiting additional factors that are required for transcriptional elongation and pre-mRNA processing as observed for other PHD finger proteins (Sims *et al.*, 2007).

Interestingly, several genes that are required for the formation of root hairs typical of Pi-deficient plants are not essential for the development of root hairs under Pi-replete conditions. For example, mutations in *AL6* cause a visible root hair phenotype only under Pi-deficient conditions. A similar Pi-dependent root hair phenotype was observed for *per1* mutants that harbour a synonymous substitution in *UBIQUITIN SPECIFIC PROTEASE14* (Li and Schmidt, 2010). This suggests that activation of a Pi-specific subset of root hair genes is required for inducing the Pi-deficiency root hair phenotype. The signalling cascade that dictates this phenotype is supposedly interlinked with Pi sensing, and its elucidation will yield valuable information on the regulation of cellular Pi homeostasis.

In the present study, we set out to dissect the role of the paralogous R3 MYB proteins CPC, ETC1 and TRY in the Pi starvation response using a transcriptional profiling approach. It is shown that the three TFs control various aspects of root hair development and Pi homeostasis in a partly non-redundant manner and are important for the coordination of cell wall-modifying enzymes, the regulation of lipid metabolism, and the orchestration of mineral nutrient homeostasis. Inferred from the function of the proteins regulated by CPC, ETC1 and TRY, we propose that these three TFs sophisticatedly control not only root hair formation, but also root-hair-unrelated processes in other types of tissues.

## Materials and methods

### Plant growth conditions

*Arabidopsis thaliana* plants were grown in a growth chamber on a solidified medium as described by Estelle and Somerville (1987). Seeds of the accession Columbia (Col-0) and *cpc* (CS6399), *etc1* (Salk_071734C) and *try* (CS6518) mutants were obtained from the Arabidopsis Biological Resource Center (Ohio State University). Seeds were surface-sterilized by immersing them in 5% (v/v) NaOCl for 5min and 70% ethanol for 7min, followed by four rinses in sterile water. Seeds were placed onto Petri dishes and kept for 1 d at 4°C in the dark, before the plates were transferred to a growth chamber and grown at 21°C under continuous illumination (50 µmol m^-2^ s^-1^; Phillips TL lamps). The medium was solidified with 0.4% Gelrite pure (Kelco), the pH was adjusted to 5.5. Low Pi conditions were obtained by growing plants on media containing 2.5 µM KH_2_PO_4_, the Pi concentration of control medium was 2.5mM KH_2_PO_4_. The lower concentration of potassium due to the reduced KH_2_PO_4_ concentration was compensated for by the addition of KCl.

### RNA-seq

Total RNA was extracted from roots using the RNeasy Plant Mini Kit (Qiagen), following the manufacturer’s instructions. For analysis, equal amounts of total RNA were collected and cDNA libraries for sequencing were prepared from total RNA following the manufacturer’s protocol (Illumina). The cDNA libraries were subsequently enriched by PCR amplification. The resulting cDNA libraries were subjected to sequencing on a single lane of an Illumina Genome Analyzer II. RNA-seq and data collection were done following the protocol of Mortazavi *et al.* (2008). The length of the cDNA library varied from 250 to 300bp with a 5′-adapter of 20bp and a 3′-adapter of 33bp at both ends.

To quantify gene expression levels, 75-mers sequences were aligned to the genomic sequence annotated in TAIR10 using the BLAT program (Kent, 2002), and RPKM (reads per kbp per million reads) values were computed using RACKJ (Read Analysis & Comparison Kit in Java, http://rackj.sourceforge.net/) software. Only those genes whose expression level in RPKM was over the square root of the mean expression value of the whole dataset (~4.5 RPKM) were considered as relevant for further analyses. Relevant differentially expressed genes were selected based on Student’s *t*-test (*P*<0.05) and 1.5-fold change in expression level between treatments.

### Bioinformatics

For gene clustering, we used the MACCU software (http://maccu.sourceforge.net/) to build co-expression networks based on co-expression relationships with a Pearson’s coefficient greater than or equal to 0.60. In order to capture the tissue-specific co-expression relationships, Pearson’s coefficients were computed based on robust multi-array averaged array data derived from leaf- and root-specific experiments for each tissue downloaded from NASCArrays (http://affymetrix.arabidopsis.info/). Visualization of the networks was performed with the Cytoscape software version 3.2.0 (http://www.cytoscape.org/).

### Real-time RT-PCR

For real-time RT-PCR, total RNA was extracted from the roots using the RNeasy Plant Mini Kit (Qiagen) and DNase treated with the Turbo DNA-free Kit (Ambion) following the manufacturer’s instructions. cDNA was synthesized using SuperScript III reverse transcription kits (Invitrogen) following the manufacturer’s instructions. Real-time PCR was performed using Power SYBR Green PCR Master Mix (Applied Biosystems) on an Applied Biosystems 7500 Fast Real-Time PCR System with programs recommended by the manufacturer. Samples were normalized first to an endogenous reference (*AtTUA*) and then the relative target gene was determined by performing a comparative ΔΔCt. The following primers were used: *AtTUA* (At5g19770) forward, GTGCTGAAGGTGGAGACGAT; reverse, AACACGAAGACCGAACGAAT.

### Measurement of root hair length and density

Confocal images with a scale bar of 100 µm at 10× resolution were used for measuring the root hair length. A ZEISS DISCOVERY V.12 microscope equipped with an ocular scale bar was used for measuring root hair density. Root hair density was measured from 2 to 6mm from the tip of the primary root. Statistical significant deviations from the wild type were determined by Student’s *t*-test. Micrographs were taken between 0 and 5mm from the tips of primary roots.

### Confocal microscopy

Roots were mounted on a glass slide with a drop of water and a coverslip, and observed with a Zeiss Axio Imager microscope equipped with a Zeiss Axiocam MRc CCD camera to capture root hair pictures. Roots were dipped in 10mg ml^-1^ propidiumiodide (PI) solution for 10 s and gently rinsed in water for 1min. Roots were then mounted on a glass slide as above and scanned using a confocal laser scanning microscope (Zeiss LSM510 Meta). The wavelength for excitation and emission of PI was 536 and 620nm, respectively.

### GUS staining

Root samples were fixed, dehydrated, and then embedded in Technovit 7100 (Heraeus Kulzer, Wehrheim) resin in gelatine capsules. Transverse sections (30 μm) were cut using a RM 2255 Leica microtome (Leica, Nussloch, Germany). Histochemical GUS staining was performed as described in Schmidt *et al.* (2007). Sections were dried and examined using brightfield on an Imager Z1 microscope (Zeiss, Jena, Germany).

### ICP-OES analysis

Mineral nutrient analysis was determined by inductively coupled plasma optical emission spectrometry (ICP-OES) as described in Rodríguez-Celma *et al.* (2013). Five plants were harvested per treatment and genotype.

## Results and discussion

### The root hair phenotype of *cpc*, *etc1* and *try* mutants is responsive to the Pi supply

Mutants defective in the expression of *CPC*, *ETC1* and *TRY* were analysed for their root hair phenotypes under control (Pi-replete) and low Pi conditions. All mutants have been described before (*cpc,* Wada *et al.*, 1997; *etc1,* Kirik *et al.*, 2004*a*; *try*, Wang *et al.*, 2013).

**Fig 1.**
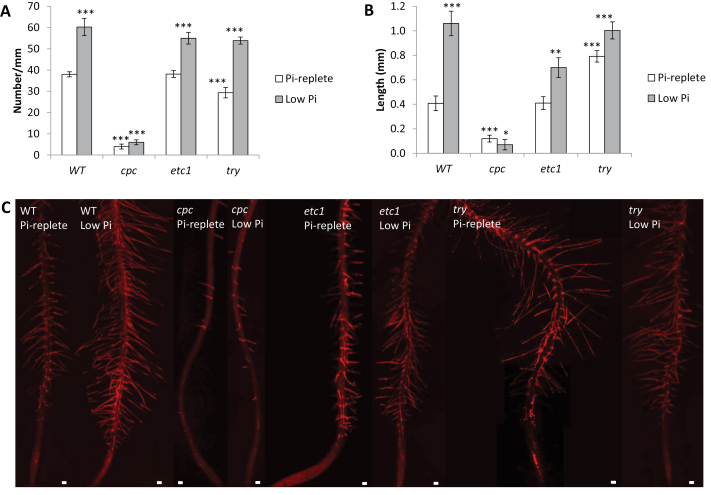
Root hair phenotypes of the wild type and mutants. (A) Root hair number. (B) Root hair length. (C) Compiled confocal micrographs of the various genotypes. The number of asterisks denotes statistically significant differences to Pi-replete wild-type plants based on Student’s t-test (* *P*<0.05, ** *P*<0.01, *** *P*<0.001). Bar, 100 μM.

As described previously, homozygous mutations in *CPC* caused a dramatic reduction in root hair density (Fig. 1A, C; Kirik *et al.*, 2004*a*). In the present investigation, root hair frequency in *cpc* was reduced by 89% and also the root hair length was strongly decreased (Fig. 1A, B). Under control (Pi-replete) conditions, no root hair phenotype was observed for *etc1* plants. In roots of *try*, the number of root hairs was reduced by 23% relative to the wild type, but root hairs were ~2-fold longer than those of wild-type plants.

In the wild type, growth on low Pi media was correlated both with increased root hair length and density (Fig. 1). An increased frequency of root hairs is a hallmark of Pi-deficient plants and has been well documented for *Arabidopsis* (Ma *et al.*, 2001; Müller and Schmidt, 2004). In agreement with previous results, the increase in root hair density in response to Pi deficiency was much less pronounced in *cpc* plants than in the wild type (Fig. 1A, C; Savage *et al.*, 2013). No increase in root hair length upon Pi starvation was observed in *cpc* plants. Roots of *etc1* did not show altered root hair density under control conditions. When grown on low Pi media, root hairs were slightly less frequent but significantly shorter than those of the wild type. Roots of *try* showed 23 and 10% reduced root hair frequency under control and low Pi conditions respectively (Fig. 1A). Under control conditions, root hairs of *try* mutants were *~*2-fold longer than those of the wild type; no difference in root hair length was observed when plants were grown on low Pi media (Fig. 1B). The difference in the root hair phenotypes between the mutants in response to Pi starvation reflects the relative importance of the three R3 MYBs for inducing root hairs typical of Pi-deficient plants.

To gain further insights into the role of the three TFs under investigation, we investigated the Pi deficiency-induced changes in the root hair phenotypes of *cpc etc1* double mutants and *TRY* overexpressing (TRY OE) lines, which were not analysed by RNA-seq. As reported previously (Savage *et al.*, 2013), the *cpc etc1* double mutant did not respond to Pi starvation with the formation of extra root hairs (Supplementary Fig. S1). In contrast, TRY OE plants showed extremely dense and long root hairs when grown on low Pi media (Supplementary Fig. S1). An independent allele of *try* (Hülskamp *et al.*, 1994) showed a behaviou similar to the allele used in the RNA-seq experiments (CS6518). Together these results support a role of all three R3 MYBs as positive regulators of the long and dense root hair phenotype of Pi-deficient plants.

### Mutations in CPC, ETC1 and TRY led to dramatic changes in the transcriptome

**Fig. 2.**
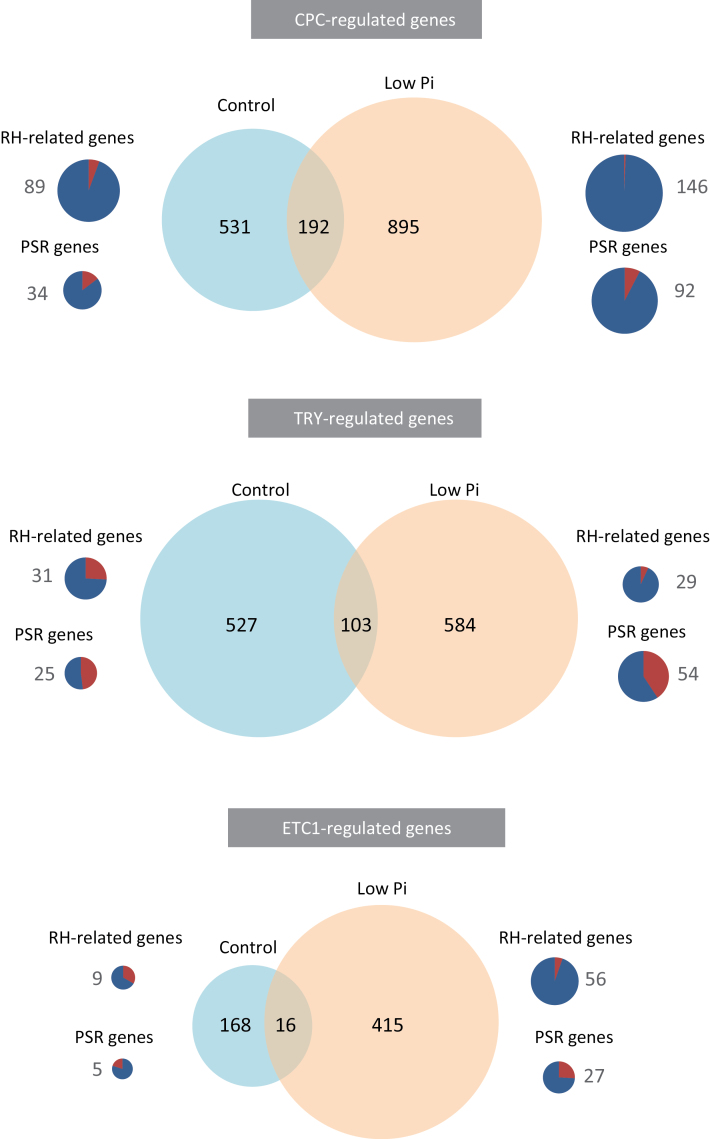
Genes differentially expressed between *cpc*, *etc1* and *try* mutants and the wild-type plants. Pie charts show genes that are preferentially expressed in root hairs (RH; Lan *et al.*, 2013) and in the phosphate starvation response (PSR) genes. Numbers represent gene counts. In the pie charts, red and blue colour represents the percentage of up- and down-regulated genes, respectively. (This figure is available in colour at JXB online.)

To identify genes that are regulated by one or more of the TFs under investigation, we surveyed the transcriptome of roots from wild-type and *cpc*, *etc1* and *try* plants using RNA-seq. Sequencing was performed in triplicates with on average ten million reads per library (Supplementary Table S1), and genes that showed a deviation in transcript abundance from the wild type greater than or equal to 1.5-fold with *P*<0.05 were defined as differentially expressed. Validation of a subset of the differentially expressed genes by qRT-PCR showed a generally good agreement between the two methods (Supplementary Fig. S2, Supplementary Table S4). For a relatively large suite of genes at least one of the three R3 MYBs was required for wild type-like expression. Under control conditions, the largest subset was dependent on *CPC* expression (723 genes), followed by *TRY* (630) and *ETC1* (184) (Fig. 2, Supplementary Table S2).

### Regulation of root hair genes by CPC, ETC1 and TRY

Consistent with their role in promoting the non-hair cell fate, the expression of *WER* and *MYB23* was higher in *cpc* and *etc1* than in wild-type plants. This increase was less pronounced in *try* mutants. In all three mutants, the negative regulator of the root hair cell fate *GL2* showed higher expression than the wild type. Also, the expression of *ROOT HAIR DEFECTIVE 6-LIKE2* (*RSL2*), encoding a functional paralogue of RSL4 (Yi *et al.*, 2010) was strongly decreased in *cpc* and *etc1*, but less so in *try* mutants.

For 120 of the 635 genes that we previously defined as preferentially expressed in root hairs (Lan *et al.*, 2013), 2-fold or greater changes in expression were observed in *cpc* plants when compared with the wild type (Supplementary Table S3). The vast majority of root hair-specific genes had lower transcript levels in the mutants than in the wild-type plants. A subset comprising 51 of these genes has been previously defined as root hair core genes (Bruex *et al.*, 2012). In *etc1* and *try*, only 12 and 15 of the differentially expressed genes, respectively, are preferentially expressed in root hairs.

In *cpc*, the expression of a suite of genes with highly root hair-specific expression (*PRP3*, *EXP7*, *EXP18*, *RHS2, RHS5, RHS7, RHS9, RHS10, RHS12-RHS14, RHS16-RHS19*) (Won *et al.*, 2009; Lan *et al.*, 2013) was repressed. Also, genes that encode proteins with experimentally validated roles in root hair formation such as the Zn^2+^ transporter ZIP3 (Lan *et al.*, 2013), RSL4 (Yi *et al.*, 2010), the phosphatidylinositol transfer protein COW1 (Grierson *et al.*, 1997), the xyloglucan endotransglycosylases XTH14 and XTH26 (Maris *et al.*, 2009), the ATPase AHA7 (Santi and Schmidt, 2009), MRH6 (Jones *et al.*, 2006), and the serine/threonine kinase IRE (Oyama *et al.*, 2002), had expression levels that were 2-fold lower in the mutant than in the wild type. The most pronounced decrease in transcripts was observed for the genes encoding the proline-rich extensin-like family protein At4g08410 and the NAD(P)-binding Rossmann-fold superfamily protein At2g30670. Also, the expression of several unknown proteins was dependent on functional CPC. Only three of these proteins (At2g34910, At3g12540 and At3g49960) have been associated with root hair development (Deal and Henikoff, 2010; Bruex *et al.*, 2012), indicating that a large number of uncharacterized proteins still await placement into the surprisingly complex puzzle of processes that control and mediate the development of root hairs. No genes with confirmed roles in root hair formation were differentially expressed in roots of *etc1* and *try*.

### Changes in R3 MYB expression upon Pi starvation

**Fig. 3:**
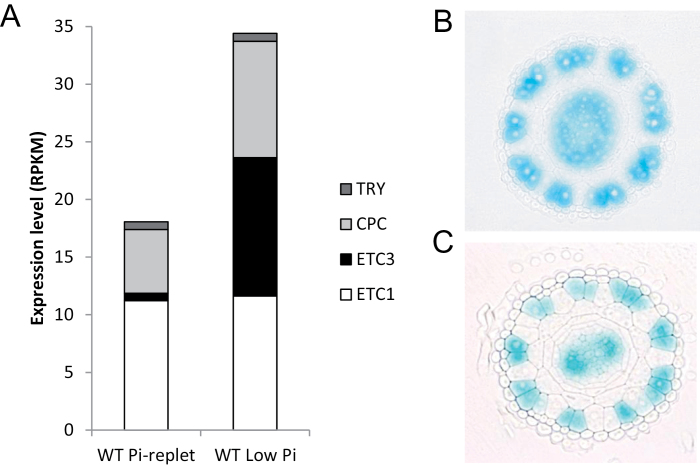
Effect of Pi deficiency on the expression of R3 MYB protein. (A) Abundance changes in the transcripts of *CPC*, *ETC1*, *ETC3* and *TRY* determined by RNA-seq analysis. Values are given in RPKM. (B, C) *CPC* promoter activity. Cross-sections are from the meristematic region of pCPC-GUS plants grown under (B) control and (C) Pi-deficient conditions. (This figure is available in colour at JXB online.)

A previous RNA-seq study revealed that of the genes that determine root epidermal cell fate only *ETC1* and *ETC3* were Pi-responsive, being significantly up-regulated upon Pi starvation (Lan *et al.*, 2012). In the present study, a relative small, non-significant increase in *ETC1* expression was observed (Fig. 3A). This difference is likely due to differences in growth conditions between the two studies [transfer to Pi-free media in Lan *et al.* (2012) versus growth on low Pi media in this study]. The expression of *CPC* increased ~2-fold when plants were grown on low Pi media, *TRY* transcript levels were not affected by the Pi regime. Notably, *ETC3* expression was low under Pi-replete conditions but was strongly induced by low Pi, resulting in a robust change in the relative abundance of the R3 MYB transcripts (Fig. 3A). Another homologue of *ETC1*, *ETC2* was not expressed in roots. Lack of detectable *ETC2* and *ETC3* transcript in (Pi-replete) roots was reported previously (Kirik *et al.*, 2004*b*; Simon *et al.*, 2007).

To investigate whether Pi deficiency alters the spatial expression pattern of *CPC*, cross-sections in the late meristematic zone of pCPC-GUS reporter lines grown under Pi-replete and low Pi conditions were analysed. In the epidermis, *GUS* expression was restricted to atrichoblasts (Fig. 3B). Similar to what have been reported previously (Wada *et al.*, 2002), in pCPC-GUS plants GUS staining was also prominent in the stele (Fig. 3B). No changes in the spatial distribution of *CPC* promoter activity were observed when plants were grown on low Pi media (Fig. 3B).

### Pi starvation induces alterations in the genes that are controlled by CPC, ETC1 and TRY

**Fig. 4.**
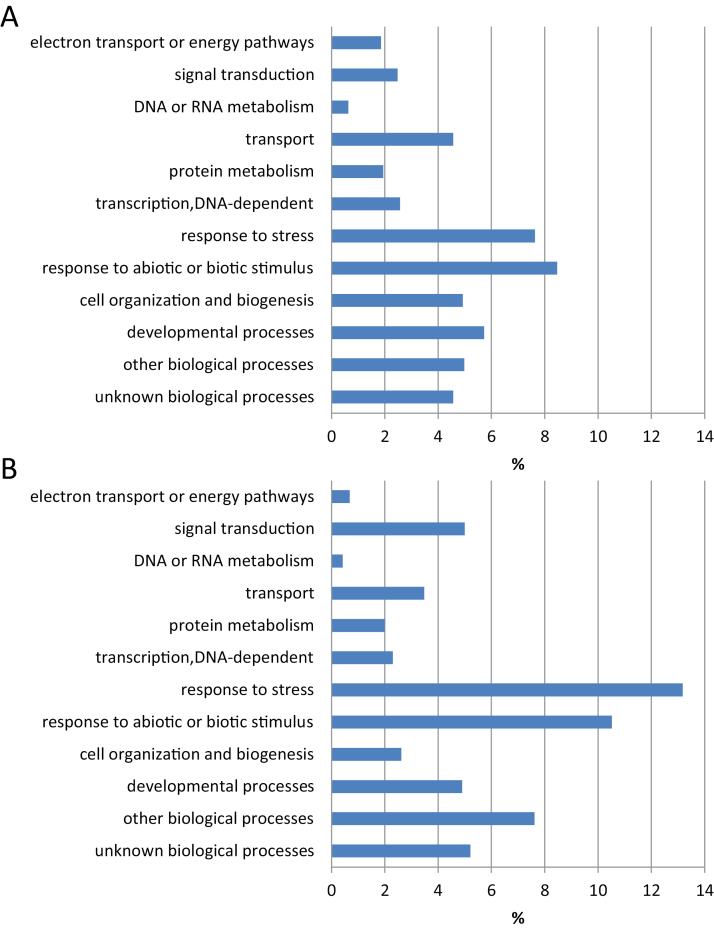
Over-represented GO categories (biological function) of genes that were differentially expressed in roots of *cpc*, *etc1* or *try* under (A) control and (B) Pi-deficient conditions. (This figure is available in colour at JXB online.)

Gene ontology (GO) enrichment analysis of genes dependent on CPC, ETC1 or TRY reveals that the biological function categories ‘cell organization and biogenesis’, ‘developmental process’, ‘DNA/RNA metabolism’ and ‘electron transport or energy pathways’ were less prominent in plants grown on low Pi, while genes belonging to the GOs ‘response to biotic or abiotic stimulus’, ‘response to stress’ and ‘signal transduction’ showed increased abundance (Fig. 4). This suggests that a dynamic shift in the roles of the three TFs under study is induced by subjecting the plants to Pi starvation.

Under low Pi conditions, the number of genes that was affected by mutations in R3 MYBs was increased by 50% for CPC, by 9% for TRY-controlled genes and by 134% for genes that are dependent of ETC1 (Fig. 2). This indicates that in particular ETC1 acquires additional functions under Pi-limited conditions. Consistent with the strong root hair phenotype of *cpc* plants under low Pi conditions, *RSL4* showed lower expression in *cpc* plants relative to the wild type under low Pi conditions (Fig. 1). *RSL4* expression was not significantly reduced under control conditions in *cpc* plants, indicating that up-regulation of *RSL4* by Pi deficiency (Yi *et al.*, 2010) is dependent on CPC and critically required for the Pi deficiency root hair phenotype. Several genes that are preferentially expressed in root hairs had message levels that were 5-fold or more lower in one or more of the mutants compared to the wild type, including the peroxidase *PRX2*, the cytochrome P450 *CYP94B3*, the squalene monooxygenase *SQP2*, and the mannose-binding lectin superfamily protein At5g49870. CYP94B3 has been associated with the oxidative catabolism of jasmonate (Kitaoka *et al.*, 2011), establishing a connection between Pi-induced root hair formation and jasmonate signalling. Transcripts of the proline-rich extensin-like protein At4g08410 and the NAD(P)-binding protein At2g30670 were also strongly down-regulated in *cpc* under both Pi-replete and low Pi conditions. The expression patterns of these genes and their regulation invite speculation on their roles in root hair elongation in particular under Pi-deficient conditions.

### Regulation of Pi-responsive Genes by CPC, ETC1 and TRY

Out of the 475 genes that were defined as being Pi-responsive (expression changes of greater than or equal to 1.5-fold with *P*<0.05), a subset of 165 was either positively or negatively affected by the loss of function of one or more of the R3 MYBs investigated. The group of Pi-responsive genes with increased expression in *cpc*, *etc1* or *try* mutants was substantially smaller than that of negatively affected genes (Fig. 2). This effect was less pronounced in roots of *try*, where almost half of the Pi-responsive genes had higher expression levels than the wild type.

Several genes encoding purple acid phosphatases (PAPs) were regulated by CPC, ETC1 or TRY (Supplementary Table S2). PAPs can release Pi from organic P due to their hydrolytic activity, thereby contributing to the acquisition of Pi from the rhizosphere. Most, but not all, of these *PAPs* were responsive to Pi. Interestingly, *PAP27,* which was down-regulated upon Pi starvation in the wild type, was co-expressed with the Zn^2+^ transporters *ZIP4*, *HMA2* and *IRT3*, all of which showed reduced transcript abundance relative to Pi-deficient wild-type plants (atted.jp). This stands in contrast to other PAPs (*e.g. PAP23*, *PAP25* and *PAP14*) that were highly up-regulated in Pi-deficient plants and co-expressed with genes involved in lipid metabolism that were strongly induced upon Pi starvation such as *PEPC1* and *MGD3*. The regulatory pattern is indicative of functional diversity of Pi-responsive PAPs, a finding that corresponds to their variable molecular and biochemical properties (Li *et al.*, 2002). The physiological functions of the R3 MYB-regulated PAPs have yet to be resolved.

### Co-expression analysis reveals functional diversity of R3 MYB-regulated genes

**Fig. 5.**
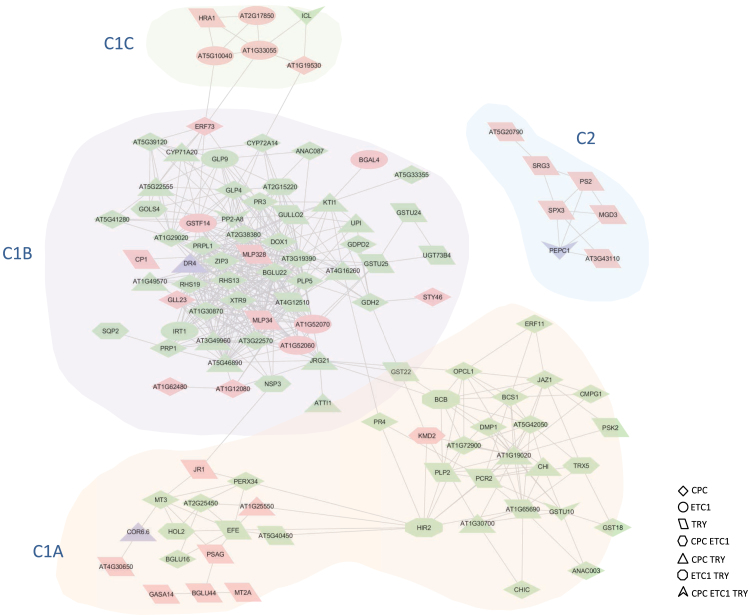
Co-expression network of PSR genes that were differentially expressed in roots of *cpc*, *etc1* and *try*. Genes were clustered based on their co-expression relationships with a Pearson’s coefficient of ≥0.60 using the MACCU software package. Pink nodes denote genes that were up-regulated in the mutants, green nodes indicate genes with decreased transcript abundance and purple nodes represent genes that are regulated in opposite directions in different mutants.

A custom-made co-expression network of the Pi-responsive genes that are dependent on at least one of the TFs under study was constructed using the MACCU toolbox, computed based on robust multi-array averaged array data derived from root-specific experiments for each tissue downloaded from NASCArrays (http://affymetrix.arabidopsis.info/; Lin *et al.*, 2011) with a Pearson correlation coefficient cutoff *P*≤0.6. This procedure yielded one large cluster (C1) that could be subdivided into three sub-clusters (C1A-C1C) and one smaller cluster (C2) (Fig. 5).

Sub-cluster 1 (C1A) contains several genes involved in redox homeostasis, with specific roles in cell death (e.g. *HIR2*, At5g42050, At1g72900), response to oxidative stress (*GASA14*, *PCR2*, *ATBCB*, At1g19020), oxidative burst (*PERX34*, *GMPG1*), and prevention of oxidative damage (*MT3*, *MT2A*). This group of genes was mostly down-regulated in *cpc* plants under low Pi conditions. *KISS ME DEADLY2* (*KMD2*), encoding a subunit of the SCF ubiquitin ligase complex that negatively regulates the cytokinin response, had increased transcript levels in *cpc* and *etc1* under low Pi conditions, indicative of a role of cytokinin in the Pi starvation response. Crosstalk between cytokinin, sugar and Pi starvation signalling has been reported (Martín *et al.*, 2000; Franco-Zorilla *et al.*, 2005; Rouached *et al.*, 2010). Pi starvation decreases cytokinin levels (Horgan and Wareing, 1980) by repressing the expression of cytokinin-related genes (Hammond *et al.*, 2003; Misson *et al.*, 2005; Morcuende *et al.*, 2007). *KMD2* was down-regulated in the wild type upon Pi starvation but had higher expression levels in *cpc* and *etc1* plants. These results are indicative of an involvement of the R3 MYBs under study in the interaction between cytokinin and Pi starvation signalling.

Sub-cluster C1B includes several genes related to the response to biotic and abiotic stress, with an overrepresentation of proteins with predicted extracellular localization. Several of these genes are preferentially expressed in root hairs and encode proteins that are involved in cell elongation (*XTR9*, *PRP1, PRPL1*, *RHS13*, *RHS19* and the peroxidase At1g30870). One of these genes, the proline-rich protein-like *PRPL1*, had lower transcript abundance in *cpc* mutants both under Pi-replete and low Pi conditions. Similar to PRP1 and PRP3, two key players in root hair differentiation that cross-connect cell wall components (Bernhardt and Tierney, 2000), the expression of *PRPL1* is restricted to trichoblasts and has been related to the elongation of root hairs (Boron *et al.*, 2014). Also the Zn^2+^/Fe^2+^ transporters *IRT1* and *ZIP3* are within this cluster. Most of the genes were down-regulated in the mutants, in particular in *cpc* and were more so under low Pi conditions.

Another group of genes in this cluster is related to lipid metabolism, comprising the patatin-related phospholipase A-encoding gene *PLP5*, two lipid-transfer proteins with undefined function (At3g22570 and At5g46890), a GDSL-like lipase (*GLL23*) and the glycerophosphodiester phosphodiesterase *GDPD2*, indicative of a link between lipid metabolism and root hair elongation. The expression of most of these genes is dependent on functional CPC. Also for these genes, the difference in transcript levels was more pronounced when plants were grown on low Pi media. A smaller sub-cluster of C1, C1C, contains five hypoxia-related genes that were all up-regulated in the mutants.

An unconnected cluster (C3) is composed of seven genes involved in lipid metabolism that were all strongly up-regulated upon Pi starvation in the wild type. The expression of several of these genes was increased in *try* mutants. A similar pattern was observed for two other genes in this cluster, encoding the TF *SPX3* and two unknown proteins (At5g20790 and At3g43110), suggesting that these three genes also participate in Pi starvation-induced reprogramming of the lipid metabolism. Interestingly, one of these genes, the pyrophosphate-dependent phosphatase *PHOSPHATE STARVATION-INDUCED GENE2* (*PS2*) PS2 was identified as a putative target of AL6 indicating that root hair elongation is co-regulated with other Pi starvation responses (PSRs) (Chandrika *et al.*, 2013).

### A lipid metabolic pathway partly controlled by CPC, ETC1 and TRY

**Fig. 6.**
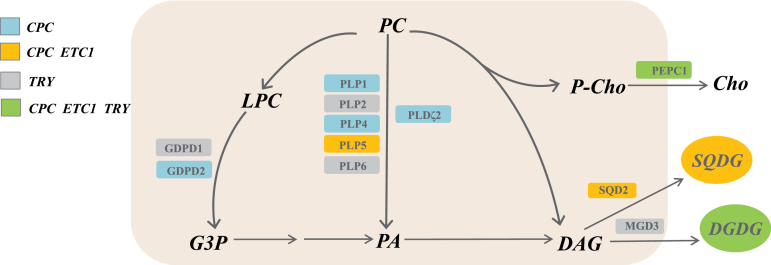
Scheme depicting the regulation and putative roles of genes involved in membrane lipid remodelling by CPC, ETC1 and TRY in Pi-deficient *Arabidopsis* roots. PC, phosphatidylcholine; LPC, lysophosphatidylcholine; G3P, glycerol-3-phosphate; PA, phosphatidic acid; P-Cho, phosphocholine; Cho, cholin; DAG, diacylglycerol. (This figure is available in colour at JXB online.)

A large percentage of Pi is recycled during Pi starvation by replacing PLs in membranes with the galactolipid digalactosyldiacylglycerol (DGDG) and a sulfolipid, sulfoquinovosyldiacylglycerol (SQDG) (‘membrane lipid remodelling’; Nakamura *et al.*, 2009; Nakamura *et al.* 2014). The current analysis suggests that membrane lipid remodelling is partly or chiefly controlled by the three R3 MYBs (see above). R3 MYB-controlled steps in a putative metabolic pathway that ultimately leads to the liberation of Pi from phospholipids (PLs) and substitution of PLs by DGDG and SQDG are depicted in Fig. 6.

PHOSPHOLIPASE D ZETA 2 (PLDζ2) has been implicated in the hydrolysis of PLs using phosphatidylcholine (PC) and phosphatidylethanolamine (PE) as substrates to produce phosphatidic acid (PA) (Cruz-Ramirez *et al.*, 2006). Full induction of PLDζ2 upon Pi starvation was dependent on functional CPC. Interestingly, PLDζ2 is also required for root hair elongation in response to Pi starvation (Li *et al.* 2006). PA could then be converted to diacylglycerol (DAG) and used as substrate for DGDG synthesis via MGD3 and for the synthesis of SQDG via SQD2. Both enzymes are strongly induced in response to Pi starvation. PEPC1 is supposedly involved in the hydrolization of phosphocholine (PCho) (May *et al.*, 2012). *PEPC1* transcript abundance was also increased in plants grown on low Pi media.

Patatin-related phospholipases A (PLPs) have been implicated in cell elongation and auxin signalling (Ryu, 2004; Rietz *et al.*, 2010), but have not been assigned a clearly defined role in the PSR. Five of the ten *Arabidopsis PLP* family members were partly regulated either by CPC, ETC1 or TRY (Fig. 5). Interestingly, alterations in *PLP4* expression affected several traits that are also responsive to Pi starvation, including changes in lipid levels and composition, primary root length and root hair elongation (Rietz *et al.*, 2010), suggesting an involvement of PLP4 and possibly other PLPs in the PSR. Out of the five *PLPs* that were de-regulated by mutations in *CPC*, *ETC1* or *TRY*, four were up-regulated upon Pi starvation. PLPs could hydrolyse PC to form lysophosphatidylcholine (LPC), which can then be converted to glycerol-3-phosphate (G3P) by GDPD1 and GDPD2 (Fig. 6).

Interestingly, PLD-derived PA has been shown to interact with WER, promoting its nuclear localization (Yao *et al.*, 2013). This finding establishes a solid mechanistic connection between lipid metabolism and root hair cell fate. Competition between different MYBs for PA binding and controlled nuclear localization of TFs represents a possible mechanism for the modulation of root hair formation and elongation in response to environmental signals.

With the exception of *PLP6*, all genes shown in Fig. 6 are only dependent on CPC, ETC1 or TRY only when grown on low Pi media. Also of note and in contrast to what was observed in *cpc* and *etc1*, several TRY-regulated genes showed a higher transcript abundance in the mutant, indicative of a negative regulation of these genes by TRY. Thus TRY on one hand and CPC and ETC1 on the other appear to play antagonistic roles in membrane lipid remodelling.

### CPC, ETC1 and TRY may regulate the activity of Pi-responsive genes partly via natural antisense transcripts

In *cpc*, *etc1* and *try* plants, several potential natural antisense transcripts (NATs) accumulated differentially between the mutants and the wild type (Table 1). Natural antisense genes are transcribed from the opposite strand of protein-coding genes and overlap in part with sense RNA, regulating sense gene expression, transcript processing, translation or degradation of sense RNA via their expression. Expression of NATs can ‘rewire’ regulatory networks by changing positive regulators into repressors through the expression of the respective antisense RNA (Pelechano and Steinmetz, 2013).

**Table 1.** Putative antisense genes controlled by CPC, ETC1 and TRY

**Antisense gene**	**Overlaps with**	**Description**	**Fold-change**
*etc1* Pi-replete			
At2g35738	At2g35740	INOSITOL TRANSPORTER 3 (INT3)	0.52
*etc1* low Pi			
At1g74205	At1g74210	GLYCEROPHOSPHODIESTER PHOSPHODIESTERASE 5 (GDPD5)	1.69
At4g26795	At4g26790	GDSL-like lipase/acylhydrolase superfamily protein	2.18
At1g60505	At1g60510	DYNAMIN RELATED PROTEIN 4D (DRP4D)	1.90
*cpc* Pi-replete			
At1g72852	At1g72850	Disease resistance protein	0.42
At1g67365	At1g67370	ASYNAPTIC 1 (ASY1)	0.33
At2g35738	At2g35740	INOSITOL TRANSPORTER 3 (INT3)	0.35
At5g07152	At5g07150	Leucine-rich repeat protein kinase family protein	2.42
*cpc* low Pi			
At4g27852	At4g27850; At4g27860	Glycine-rich protein family; MEMBRANE OF ER BODY 1 (MEB1)	0.35
At1g03545	At1g03550	SECRETORY CARRIER MEMBRANE PROTEIN 4 (SCAMP4)	2.98
At1g07128	At1g07130	STN1	0.18
At3g22072	At3g22070	Proline-rich family protein	∞
At1g28685	At1g28680	HXXXD-type acyl-transferase family protein	0.14
At3g21755	At3g21760	HYPOSTATIN RESISTANCE 1 (HYR1)	7.12
At3g48115	At3g48120	Unknown protein	0.56
At2g22821	At2g22820	Unknown protein	0.22
*try* low Pi			
At1g60505	At1g60510	DYNAMIN RELATED PROTEIN 4D (DRP4D)	1.88
At4g26795	At4g26790	GDSL-like lipase/acylhydrolase superfamily protein	2.99
At4g31398	At4g31400	CHROMOSOME TRANSMISSION FIDELITY 7 (CTF7)	0.14
At3g21755	At3g21760	HYPOSTATIN RESISTANCE 1 (HYR1)	5.46
At4g13700	At1g44120	CELLULOSE SYNTHASE INTERACTIVE 2 (CSI2)	1.60
At1g56165	At1g56160	MYB72	0.35
*try* Pi-replete			
At2g46572	At2g46570	LACCASE 6 (LAC6)	2.62
At3g24518	At3g24520	HEAT SHOCK TRANSCRIPTION FACTOR C1 (HSFC1)	0.40
At5g01175	At5g01180	PEPTIDE TRANSPORTER 5 (PTR5)	1.59
At1g28685	At1g28680	HXXXD-type acyl-transferase family protein	0.38
At2g35738	At2g35740	INOSITOL TRANSPORTER 3 (INT3)	0.49
At2g35637	At2g35640	Homeodomain-like superfamily protein	0.43
At1g60525	At1g60530	DYNAMIN RELATED PROTEIN 4A (DRP4D)	1.61

In *etc1* and *cpc* but not in *try* plants, differential expression of antisense genes was more frequent when plants were grown under low Pi conditions. Among the putatively regulated genes, two are encoding proteins involved in lipid metabolism (GDPD5 and the GDSL-like lipase At4g26790); the respective antisense genes were regulated by ETC1 and TRY. A potential antisense gene that is regulated by CPC under low Pi conditions overlaps with the proline-rich protein At3g22070. At3g22070 is co-expressed with several important genes in root hair formation such as *LRX2*, *LRX3* and *JKD* (atted.jp) and may thus be involved in root hair elongation in response to Pi starvation. Another CPC-regulated NAT overlaps with the secretory carrier membrane protein SCAMP4, a node of a gene regulatory network that controls root hair development (Bruex *et al.*, 2012). The antisense gene that potentially regulates the inositol transporter *INT3* was controlled by all TFs under study. Inositol can bind Pi to and store Pi as phytate (InsP6). Inositol polyphosphate kinases have been implicated in root hair elongation and Pi sensing (Stevensen-Paulik *et al.*, 2005). Thus, the control of *INT3* by NATs may have impact on cellular Pi homeostasis and may indirectly influence root hair elongation via alterations of the plant’s Pi status.

The most pronounced effect of mutations in R3 MYBs was observed for At3g21755, a putative antisense gene of *HYR1*. *HYR1* was highly up-regulated in *cpc* and *try* mutants in both growth types (Pi-replete and low Pi), but was more so in Pi starved plants (more than 7-fold up in *cpc* and more than 5-fold up in *try*). *HYR1* encodes a UDP glycosyltransferase that confers resistance against the cell expansion inhibitor hypostatin via glucosylation (Zhao *et al.*, 2007). The role of HYR1 in Pi homeostasis in roots and its regulation awaits further characterization.

### CPC and ETC1 participate in the regulation of cellular zinc and Pi homeostasis

**Fig. 7.**
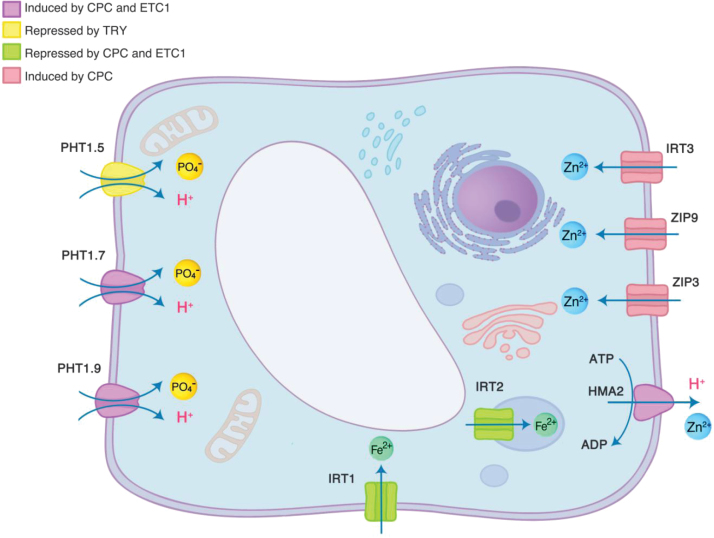
Regulation of transition metal transporters by CPC, ETC1 and TRY. The transport proteins are likely localized in different cell types and tissues.

To further decipher processes that are regulated by the three TFs under control and/or low Pi conditions, we considered genes in which the ratio (wild type low Pi/wild type control)/(mutant low Pi/mutant control) was more than 2-fold changed. Interestingly, several genes encoding transition metal and Pi transporters regulated by CPC and ETC1 fulfilled this criterion (Fig. 7). In particular, genes encoding plasma membrane (PM)-bound Fe^2+^/Zn^2+^ transporters were positively regulated either by CPC alone (*ZIP3*, *ZIP9*), or by both CPC and ETC1 (*HMA2*). In the wild type, the expression of Zn^2+^ transporters was strongly reduced upon Pi starvation, a response that was less pronounced in *cpc* and *etc1* mutants. In addition, under control conditions, expression levels of these genes were 2–3-fold lower in the mutants than in the wild type. In contrast, the expression of the Fe^2+^ transporters *IRT1* and *IRT2* was higher in *cpc* and *etc1* mutants than in the wild type when grown on Pi-replete media. Pi starvation leads to an increase in Fe concentrations for reasons that are still under debate, causing a pronounced down-regulation of the Fe^2+^ transporters (Misson *et al.*, 2005; Lan *et al.*, 2012). This down-regulation was more pronounced in *cpc* and *etc1* mutants. It thus appears that under Pi-replete conditions the expression of Zn^2+^ transporters is reduced in the mutants relative to the wild type, while that of Fe^2+^ transporters was higher. Under low Pi conditions, this relationship is reversed, i.e. a slightly higher (but statistically insignificant) expression of Zn^2+^ transporters and a lower expression of Fe^2+^transporters in the mutants. In support of an altered Zn^2+^ homeostasis in response to Pi starvation, the PM-bound Zn^2+^ exporter *PCR2* was highly induced upon Pi starvation in roots of wild-type plants. In all three mutants, the induction of *PCR2* was lower than in the wild type, but the difference did not reach the 2-fold threshold used here.

The plasma membrane (PM)-localized Pi transporters *PHT1;5*, *PHT1;7* and *PHT1;9* were strongly induced in the wild type upon Pi starvation, but this increase was much less pronounced in *cpc* and *etc1* mutants. In *try* roots, the (wild type low Pi/wild type control)/(mutant low Pi/mutant control) ratio of *PHT1;5* expression was decreased, due to a lower expression of *PHT1;5* (0.36-fold) under control conditions and a slightly increased expression under low Pi conditions when compared with the wild type.

The transporters depicted in Fig. 7 may not be localized in the same cell type and are summarized here in a single cell for simplicity. With the exception of IRT2, which is expressed in internal membranes (Vert *et al.*, 2009), all transporters have a predicted or experimentally validated localization on the PM. IRT1 is preferentially expressed in root epidermal cells (Vert *et al.*, 2002). Governed by its low substrate specificity, IRT1 transports other metal ions such as Zn^2+^ and Mn^2+^ beside Fe^2+^ (Korshunova *et al.*, 1999). The primary active Zn^2+^ pump HMA2 catalyses the loading of Zn^2+^ into the xylem and is predominantly expressed in the vasculature (Hussain *et al.*, 2004). Based on a similar expression pattern, IRT3 has been associated with xylem unloading and/or phloem loading (Lin *et al.*, 2009). While a role of transition metal homeostasis in root hair formation remains to be demonstrated, the results make it tempting to speculate that the concentration, distribution or relative concentration in relation of Zn to other transition metals is critical for the differentiation of trichoblasts. This assumption is supported by the root hair phenotype of *zip3* mutants (Lan *et al.*, 2013). The sophisticated, Pi-responsive control of cellular Zn levels is dependent on CPC and partly on ETC1. Whether the regulation of these transporters by R3 MYBs is direct or indirect remains elusive.

Also the Pi transporters that were affected by the mutations in CPC, ETC1 and TRY are most likely expressed in different root tissues. While PHT1;9 was implicated in Pi acquisition from the soil-root interphase under conditions of Pi starvation (Remy *et al.*, 2012), PHT1;5 was shown to be involved in P allocation to shoots (Nagarajan *et al.*, 2011), indicative of a localization in the stele. The regulation of genes primarily expressed in the epidermis and the stele is in line with the expression pattern of *CPC* (Fig. 3). Interestingly, ectopic expression of *PHT1;5* resulted in increased root hair formation and reduced primary root growth. *PHT1;5* transcripts were decreased in *WRKY75* RNAi lines (Nagarajan *et al.*, 2011). WRKY75 non-autonomously regulates the expression of *CPC* and indirectly that of *GL2*, thereby altering the number of hairs in non-hair cell files (Rishmawi *et al.*, 2014). *WRKY75* is induced upon Pi starvation; suppression of *WRKY75* significantly reduced root hair density both under control and Pi-deficient conditions (Devaiah *et al.*, 2007). Together these results suggest that WRKY75 might affect root hair formation and Pi homeostasis via controlling the expression of *CPC.*

## Conclusions

The current study shows that the three TFs under study directly or indirectly control a plethora of processes mediated by >2000 genes, including cell wall remodelling, Pi transport and storage, transition metal homeostasis, hormone signalling and lipid metabolism. This diversity is surprising since, as positive regulators of the root hair cell fate, CPC, ETC1 and TRY supposedly have targets that are chiefly associated with processes involved in root hair differentiation. It should also be noted that the number of genes that are regulated by CPC, ETC1 and TRY might be underestimated by the current approach since genes that are regulated in a fully fashioned manner are not detected.

The genes that are affected by mutations in the TFs investigated shifted in response to Pi starvation towards GO categories that are related to stress response and signal transduction, indicating an extension of their role to confer phenotypic plasticity. This shift may be caused by changes in R3 MYB expression levels, interaction with other proteins, competition for promoter binding sites or other factors. A tempting scenario implies regulation of the nuclear localization of R3 MYBs and WER/MYB23 via differential binding to PA (or other lipids), which would positively or negatively regulate root hair cell fate and also control several processes that adapt plants to low Pi availability. Dynamic shifts in the nuclear localization of particular MYBs would not affect the steady-state abundance of their transcripts, but may have strong impacts on downstream transcription.

The current dataset is also indicative of a sophisticated interplay of the three TFs, contrary to an understanding of a largely redundant functional relationship. This interplay becomes apparent particularly during Pi starvation, where the relative abundance of the three R3 MYBs is massively altered to favour ETC1, ETC3 and CPC while TRY abundance remains un-responsive to the Pi supply. A possible regulatory mechanism implies that membrane lipid remodelling is calibrated by the relative abundance of ETC3 and CPC as positive regulators and the negative regulator TRY; a concept that awaits experimental validation.

Our data further reveal that root hair development under both control and Pi-deficient conditions may be intertwined with various processes located in different tissues, which may directly or indirectly affect the differentiation of epidermal cells in a feedback mechanism. For example, the regulation of Pi and Zn^2+^ distribution among different tissues or organs may alter cellular nutrient homeostasis, which in turn has consequences for epidermal cell differentiation. Another example is membrane lipid remodelling that may also affect root hair development indirectly by altering the level of free Pi and thereby the overall Pi status of the plant and/or by its impact on Pi signalling. Also, changes in the storage of Pi by the regulation of inositol transport may influence root hair formation. Similar to the plant hormones auxin and ethylene that act downstream of the cell specification cascade (Masucci and Schiefelbein, 1996), other factors such as the internal concentration of transition metals may have direct consequences for the phenotype. In contrast to hormones, which act on the cell fate via a route that is independent of the decisions made upstream, our data reveal that R3 MYBs control a variety of physiological processes, which may in turn affect epidermal patterning.

In summary, the current survey reveals several novel candidates that may play important yet undiscovered roles in root hair formation. The data obtained here also challenge the generalization of genetic redundancy of paralogous genes by demonstrating that such apparent redundancy, while true under controlled conditions, may turn into a complex interplay of gene control when environmental factors dictate adaptive alterations of metabolic and developmental programmes.

## Supplementary data

Supplementary data are available at *JBX* online.

Supplementary Figure S1. Root hair phenotypes of the *cpc etc1* double mutant, *try*, and an *TRY* overexpressing line.

Supplementary Figure S2. Validation of differentially expressed genes by qRT-PCR. Supplementary Table S1. Number of reads of the various libraries.

Supplementary Table S2. Ratios [low Pi (LP)/Pi-replete (ES)] of differentially expressed genes in the wild type and the mutants investigated.

Supplementary Table S3. Genes that are more than 2-fold differentially expressed between wild-type plants and *cpc*, *etc1* and *try* mutants.

Supplementary Table S4. Primers used for the qRT-PCR experiments shown in Figure S2.

Supplementary Data
