# Supplementary material for: The paralogous R3 MYB proteins CAPRICE, TRIPTYCHON and ENHANCER OF TRY AND CPC1 play pleiotropic and partly non-redundant roles in the phosphate starvation response of Arabidopsis roots
Source: J Exp Bot. 2015 May 28;66(15):4821–34. doi: 10.1093/jxb/erv259 (PMC4507782; doi:10.1093/jxb/erv259)
Supplement: Supplementary Data [file supp_66_15_4821__index.html]

The paralogous R3 MYB proteins CAPRICE, TRIPTYCHON and ENHANCER OF TRY AND CPC1 play pleiotropic and partly non-redundant roles in the phosphate starvation response of Arabidopsis roots — The paralogous R3 MYB proteins CAPRICE, TRIPTYCHON and ENHANCER OF TRY AND CPC1 play pleiotropic and partly non-redundant roles in the phosphate starvation response of Arabidopsis roots — Supplementary Data 

# The paralogous R3 MYB proteins CAPRICE, TRIPTYCHON and ENHANCER OF TRY AND CPC1 play pleiotropic and partly non-redundant roles in the phosphate starvation response of *Arabidopsis* roots

## Supplementary Data

Data files

- Supplementary Data - Supplementary Data
- Supplementary Data - Supplementary Data
