# Supplementary material for: The paralogous R3 MYB proteins CAPRICE, TRIPTYCHON and ENHANCER OF TRY AND CPC1 play pleiotropic and partly non-redundant roles in the phosphate starvation response of Arabidopsis roots
Source: J Exp Bot. 2015 May 28;66(15):4821–34. doi: 10.1093/jxb/erv259 (PMC4507782; doi:10.1093/jxb/erv259)
Supplement: Supplementary Data [file supp_erv259_jexbot147298_file002.pdf]

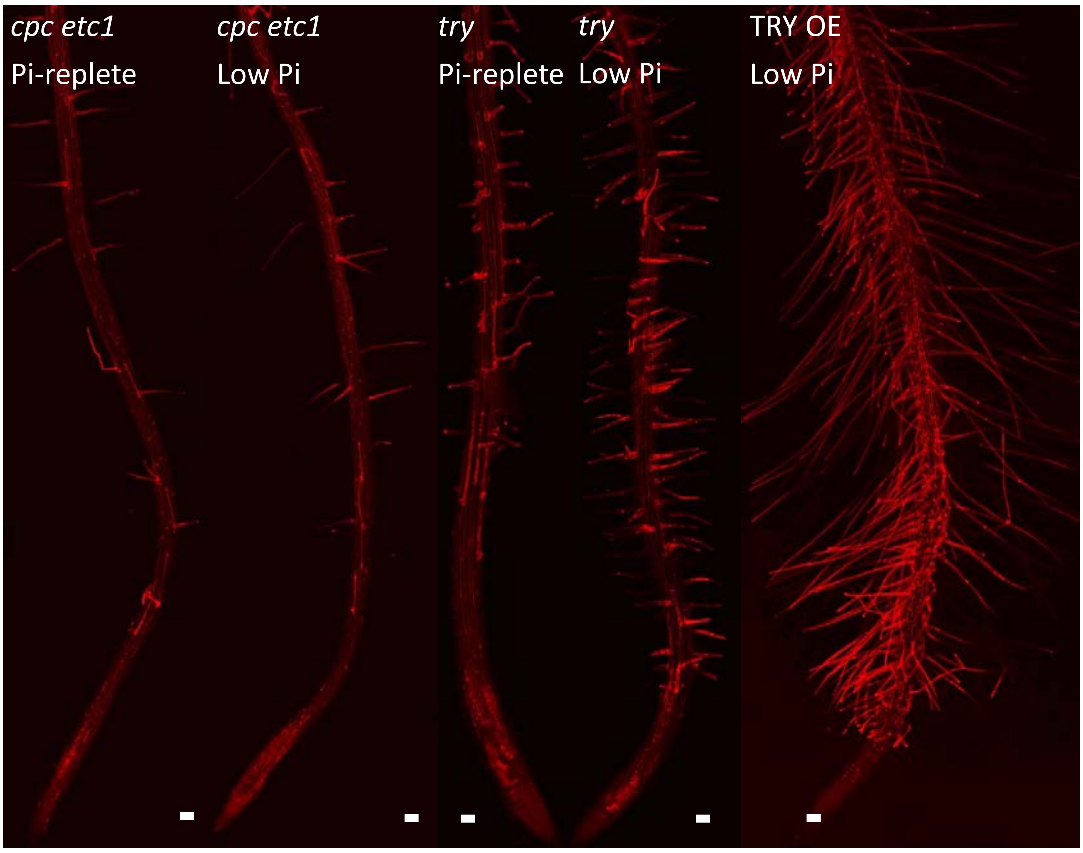


**Supplementary Figure S1.** Root hair phenotypes of the *cpc etc1* double mutant, *try*, and an *TRY* overexpressing line (TRY OE). The *try* allel shown here has been described in (Hülskamp et al., 1994). Micrographs are compiled confocal micrographs of the various genotypes. Scale bar = 100 µM.

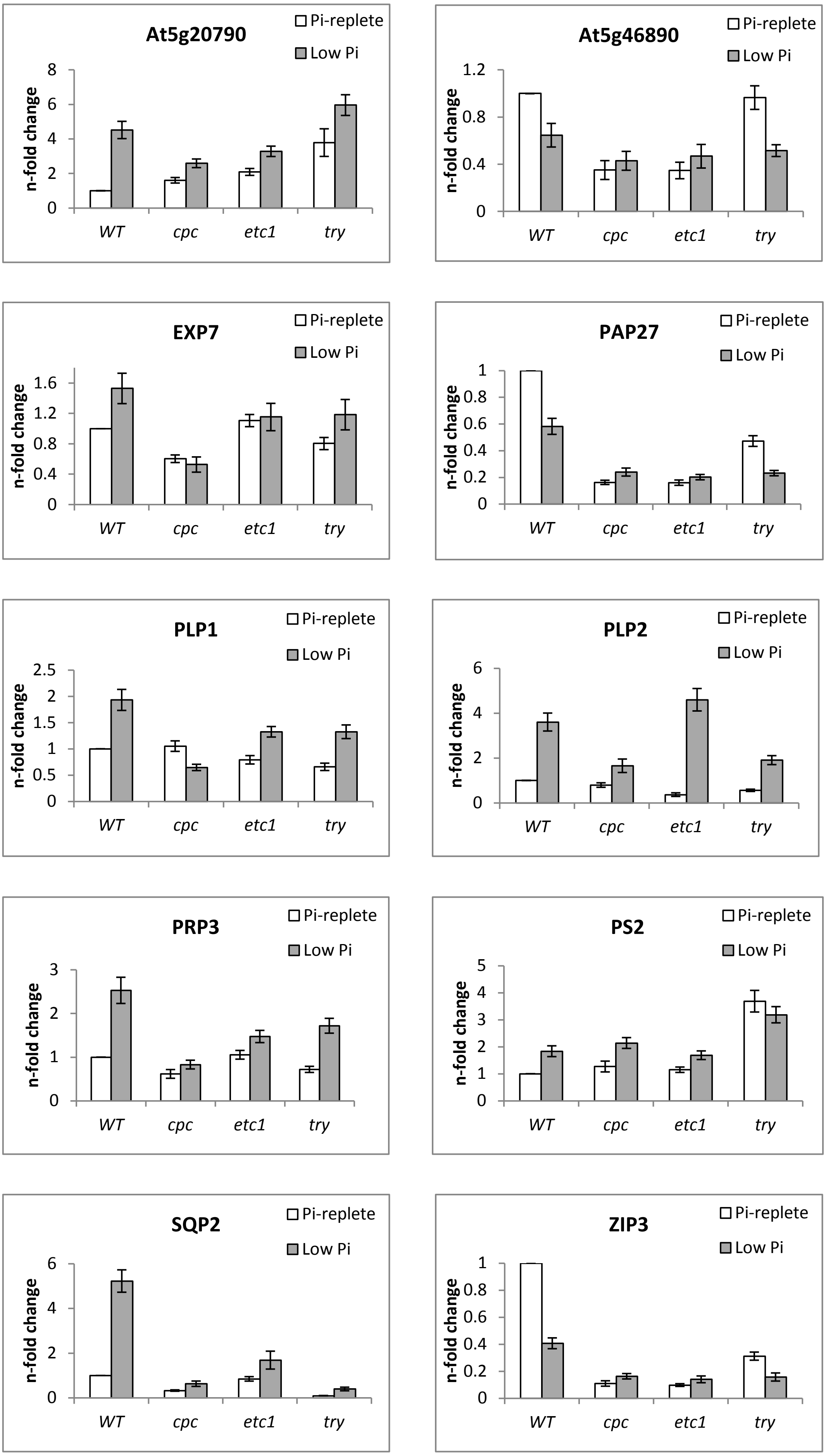


**Supplementary Figure S2.** Validation of differentially expressed genes by qRT-PCR. Experiments were run in triplicates. Error bars denote standard deviation.

**Supplementary Table S1.** Number of reads of the various libraries. WT, wild type.

| Library | Unique reads | Total reads | Unique reads/reads (%) |
|---|---:|---:|---:|
| WT Pi-replete1 | 8845094 | 9583543 | 92.29 |
| WT Pi-replete2 | 9622210 | 10395752 | 92.56 |
| WT Pi-replete3 | 10000461 | 10786076 | 92.72 |
| WT Low Pi1 | 9938714 | 10682906 | 93.03 |
| WT Low Pi2 | 9568663 | 10362704 | 92.34 |
| WT Low Pi3 | 8986142 | 9715360 | 92.49 |
| *cpc* Pi-replete1 | 9109117 | 9889849 | 92.11 |
| *cpc* Pi-replete2 | 9421707 | 10189057 | 92.47 |
| *cpc* Pi-replete3 | 8700407 | 9387797 | 92.68 |
| *cpc* Low Pi1 | 8723705 | 9440466 | 92.41 |
| *cpc* Low Pi2 | 9255124 | 9960043 | 92.92 |
| *cpc* Low Pi3 | 9069630 | 9797352 | 92.57 |
| *etc1* Pi-replete1 | 9000586 | 9670730 | 93.07 |
| *etc1* Pi-replete2 | 9151739 | 9885250 | 92.58 |
| *etc1* Pi-replete3 | 9432235 | 10159786 | 92.84 |
| *etc1* Low Pi1 | 8902453 | 9625233 | 92.49 |
| *etc1* Low Pi2 | 9186993 | 9876860 | 93.02 |
| *etc1* Low Pi3 | 9392098 | 10100167 | 92.99 |
| *try* Pi-replete1 | 8708928 | 9481538 | 91.85 |
| *try* Pi-replete2 | 9662148 | 10437464 | 92.57 |
| *try* Pi-replete3 | 9274922 | 10048367 | 92.30 |
| *try* Low Pi1 | 9108422 | 9806155 | 92.88 |
| *try* Low Pi2 | 9738636 | 10493338 | 92.81 |
| *try* Low Pi3 | 9248350 | 9962855 | 92.83 |

**Supplementary Table S3.** Genes that are more than 2-fold differentially expressed between wild-type plants and *cpc*, *etc1* and *try* mutants. n.c., not changed.

| Locus | Name | Biol. process | Fold-change (Control) | Fold-change (Low Pi) |
|---|---|---|---|---|
| *CPC-regulated genes* | | | | |
| AT1G05240 | Peroxidase superfamily protein | Response to oxidative stress | 0.40 | n.c. |
| AT1G05250 | PEROXIDASE 2 (PRX2) | Cell wall organization | n.c. | 0.04 |
| AT1G05340 | Unknown protein | Response to chitin | 0.43 | n.c. |
| AT1G06923 | Unknown protein | Unknown | 0.49 | 0.38 |
| AT1G12560 | EXPANSIN 7 (EXP7) | Root hair elongation | 0.44 | 0.33 |
| AT1G12950 | ROOT HAIR SPECIFIC 2 (RSH2) | Root hair cell differentiation | n.c. | 0.49 |
| AT1G15010 | Unknown protein | Defense response | 0.38 | 0.33 |
| AT1G19200 | Domain of unknown function (DUF581) | Response to phosphate starvation | n.c. | 0.48 |
| AT1G20310 | Unknown protein | Protein targeting to membrane | 0.42 | 0.30 |
| AT1G24320 | Six-hairpin glycosidases superfamily protein | Qligosaccharide metabolic process | n.c. | 0.37 |
| AT1G27740 | ROOT HAIR DEFECTIVE 6-LIKE 4 (RSL4) | Root hair cell differentiation | n.c. | 0.49 |
| AT1G30870 | Peroxidase superfamily protein | Trichoblast differentiation | n.c. | 0.48 |
| AT1G30990 | Polyketide cyclase/dehydrase and lipid transport superfamily protein | Defense response | n.c. | 0.43 |
| AT1G33090 | MATE efflux family protein | Transmembrane transport | 0.29 | 0.40 |
| AT1G34760 | ROOT HAIR SPECIFIC 5 (RHS5) | Response to blue light | n.c. | 0.31 |
| AT1G45145 | THIOREDOXIN H-TYPE 5 (TRX5) | Defense response | n.c. | 0.45 |
| AT1G48640 | Transmembrane amino acid transporter family protein | Amino acid transport | 0.44 | n.c. |
| AT1G48930 | GLYCOSYL HYDROLASE9 C1 (GH9C1) | Trichoblast differentiation | n.c. | 0.41 |
| AT1G52240 | ROP GUANINE NUCLEOTIDE EXCHANGE FACTOR 11 (ROPGEF11) | Root hair development | n.c. | 0.41 |
| AT1G52660 | P-loop containing nucleoside triphosphate hydrolases superfamily protein | Defense response | 0.49 | 0.39 |
| AT1G54970 | ROOT HAIR SPECIFIC 7 (RHS7) | Trichoblast differentiation | n.c. | 0.35 |
| AT1G56660 | Unknown protein | Unknown | n.c. | 0.46 |
| AT1G58270 | ZW9 | Unknown | 0.42 | 0.24 |
| AT1G62980 | EXPANSIN 18 (EXP18) | Cell wall modification | 0.45 | 0.32 |
| AT1G66160 | CYS, MET, PRO, AND GLY PROTEIN 1 (CMPG1) | Defense response | n.c. | 0.41 |
| AT1G69240 | ROOT HAIR SPECIFIC 9 (RHS9) | Hydrolase activity | n.c. | 0.46 |
| AT1G70460 | ROOT HAIR SPECIFIC 10 (RHS10) | Root hair cell differentiation | 0.46 | 0.39 |

| | | | | |
|---|---|---|---|---|
| AT1G74830 | Domain of unknown function (DUF593) | Unknown | n.c. | 0.39 |
| AT1G78070 | WD40 repeat-like superfamily protein | Unknown | n.c. | 0.48 |
| AT2G03720 | MORPHOGENESIS OF ROOT HAIR 6 (MRH6) | Root hair development | n.c. | 0.44 |
| AT2G05160 | CCCH-type zinc finger family protein | Nucleic acid binding | 0.49 | 0.45 |
| AT2G17660 | RPM1-interacting protein 4 (RIN4) family protein | Defense response | 0.47 | 0.33 |
| AT2G20880 | ERF DOMAIN53 (ERF53) | Response to salt stress | 2.80 | n.c. |
| AT2G22290 | RAB GTPASE HOMOLOG H1D (RABH1D) | Cell wall modification | n.c. | 0.30 |
| AT2G24980 | EXTENSIN 6 (EXT6) | Cell wall organization | 0.39 | 0.46 |
| AT2G25240 | CONSERVED IN CILIATED SPECIES AND IN THE LAND PLANTS 3 (CCP3) | Regulation of proteolysis | 0.36 | 0.23 |
| AT2G29620 | Unknown protein | Unknown | n.c. | 0.32 |
| AT2G30040 | MITOGEN-ACTIVATED PROTEIN KINASE KINASE KINASE 14 (MAPKKK14) | Regulation of transcription | 0.42 | n.c. |
| AT2G30395 | OVATE FAMILY PROTEIN 17 (OFP17) | Regulation of transcription | n.c. | 0.44 |
| AT2G30670 | NAD(P)-binding Rossmann-fold superfamily protein | oxidation reduction | 0.18 | 0.17 |
| AT2G32270 | ZINC TRANSPORTER 3 PRECURSOR (ZIP3) | Root hair elongation | 0.49 | n.c. |
| AT2G33460 | ROP-INTERACTIVE CRIB MOTIF-CONTAINING PROTEIN 1 (RIC1) | Pollen tube growth | 0.45 | n.c. |
| AT2G34180 | CBL-INTERACTING PROTEIN KINASE 13 (CIPK13) | Unknown | n.c. | 0.29 |
| AT2G34910 | Unknown protein | Root hair elongation | n.c. | 0.45 |
| AT2G44110 | MILDEW RESISTANCE LOCUS O 15 (MLO15) | Trichoblast differentiation | n.c. | 0.49 |
| AT2G46860 | PYROPHOSPHORYLASE 3 (PPA3) | Metabolic process | n.c. | 0.48 |
| AT2G47530 | Pollen Ole e 1 allergen and extensin family protein | Unknown | 0.44 | 0.47 |
| AT2G47540 | Pollen Ole e 1 allergen and extensin family protein | Root hair cell differentiation | n.c. | 0.48 |
| AT3G09925 | Pollen Ole e 1 allergen and extensin family protein | Unknown | n.c. | 0.46 |
| AT3G10710 | ROOT HAIR SPECIFIC 12 (RHS12) | Cell wall modification | 0.49 | 0.38 |
| AT3G12540 | Domain of unknown function (DUF547) | Root hair cell differentiation | 0.41 | 0.42 |
| AT3G16390 | NITRILE SPECIFIER PROTEIN 3 (NSP3) | Nitrile biosynthetic process | 0.50 | 0.40 |

| | | | | |
|---|---|---|---|---|
| AT3G45060 | HIGH AFFINITY NITRATE TRANSPORTER 2.6 (NRT2.6) | Nitrate transport | n.c. | 0.36 |
| AT3G47340 | GLUTAMINE-DEPENDENT ASPARAGINE SYNTHASE 1 (ASN1) | Response to sucrose starvation | n.c. | 0.31 |
| AT3G48520 | CYTOCHROME P450 FAMILY 94 SUBFAMILY B POLYPEPTIDE 3 (CYP94B3) | Response to wounding | 0.38 | 0.18 |
| AT3G49960 | Unknown protein 2x | Trichoblast differentiation | 0.38 | 0.49 |
| AT3G53150 | UDP-GLUCOSYL TRANSFERASE 73D1 (UGT73D1) | Amino acid transport | n.c. | 0.35 |
| AT3G53600 | C2H2-type zinc finger family protein | Regulation of transcription | n.c. | 0.34 |
| AT3G54870 | MORPHOGENESIS OF ROOT HAIR 2 (MRH2) | Root hair tip growth | n.c. | 0.46 |
| AT3G60280 | UCLACYANIN 3 (UCC3) | Copper ion binding | 0.45 | 0.42 |
| AT3G60330 | H(+)-ATPASE 7 (AHA7) | Cation transport | n.c. | 0.35 |
| AT3G62680 | PROLINE-RICH PROTEIN 3 (PRP3) | Trichoblast differentiation | n.c. | 0.42 |
| AT4G00680 | ACTIN DEPOLYMERIZING FACTOR 8 (ADF8) | Trichoblast differentiation | n.c. | 0.41 |
| AT4G02270 | ROOT HAIR SPECIFIC 13 (RHS13) | Trichoblast differentiation | n.c. | 0.43 |
| AT4G07960 | CELLULOSE-SYNTHASE-LIKE C12 (CSLC12) | Cellulose synthase activity | n.c. | 0.44 |
| AT4G08400 | Proline-rich extensin-like family protein | Cell wall organization | 0.46 | 0.49 |
| AT4G08410 | Proline-rich extensin-like family protein | Cell wall organization | 0.21 | 0.17 |
| AT4G19230 | CYTOCHROME P450 FAMILY 707 SUBFAMILY A POLYPEPTIDE 1 (CYP707A1) | Abscisic acid metabolic process | 0.37 | 0.43 |
| AT4G19680 | IRON REGULATED TRANSPORTER 2 (IRT2) | Response to iron starvation and nitrate starvation | n.c. | 0.33 |
| AT4G22080 | ROOT HAIR SPECIFIC 14 (RHS14) | Pectate lyase activity | 0.36 | 0.28 |
| AT4G22214 | Defensin-like (DEFL) family protein | Unknown | 0.46 | 0.28 |
| AT4G22217 | Defensin-like (DEFL) family protein | Unknown | 0.47 | 0.44 |
| AT4G24580 | ROP1 ENHANCER 1 (REN1) | Pollen tube growth | 0.43 | 0.46 |
| AT4G25790 | CAP (Cysteine-rich secretory proteins, Antigen 5, and Pathogenesis-related 1 protein) superfamily protein | Trichoblast differentiation | 0.44 | 0.49 |
| AT4G25820 | XYLOGLUCAN ENDOTRANSGLUCOSYLASE/HYDROLASE 14 (XTH14) | Trichoblast differentiation | 0.50 | 0.48 |

| | | | | |
|---|---|---|---|---|
| AT4G26770 | Phosphatidate cytidylyltransferase family protein | Phospholipid biosynthetic process | n.c. | 0.49 |
| AT4G27290 | S-locus lectin protein kinase family protein | Root hair cell differentiation | 0.46 | 0.46 |
| AT4G28850 | XYLOGLUCAN ENDOTRANSGLUCOSYLASE/HYDROLASE 26 (XTH26) | Carbohydrate metabolic process | 0.48 | 0.41 |
| AT4G29180 | ROOT HAIR SPECIFIC 16 (RHS16) | Root hair cell differentiation | 0.47 | 0.41 |
| AT4G30320 | CAP (Cysteine-rich secretory proteins, Antigen 5, and Pathogenesis-related 1 protein) superfamily protein | Trichoblast differentiation | 0.50 | 0.39 |
| AT4G34580 | CAN OF WORMS 1 (COW11) | Root hair elongation | 0.46 | 0.40 |
| AT4G35200 | Domain of unknown function (DUF241) | Unknown | 2.28 | n.c. |
| AT4G38390 | ROOT HAIR SPECIFIC 17 (RHS17) | Unknown | n.c. | 0.45 |
| AT4G39890 | RAB GTPASE HOMOLOG H1C (RABH1C) | Small GTPase mediated signal transduction | n.c. | 0.48 |
| AT5G01050 | LACCASE-9 (LAC9) | Unknown | n.c. | 0.43 |
| AT5G01100 | FRIABLE 1 (FRB1) | O-fucosyltransferase family protein | n.c. | 0.24 |
| AT5G01280 | BASIC PROLINE-RICH PROTEIN3 (BPP3) | Response to UV light | 0.43 | n.c. |
| AT5G05500 | PROLINE-RICH PROTEIN-LIKE 1 (PRPL1) | Root hair elongation | n.c. | 0.47 |
| AT5G06760 | LATE EMBRYOGENESIS ABUNDANT 4-5 (LEA4-5) | Response to osmotic stress | n.c. | 0.40 |
| AT5G11070 | Unknown protein | Response to brassinosteroid stimulus | n.c. | 0.43 |
| AT5G18910 | Protein kinase superfamily protein | Amino acid phosphorylation | 0.31 | 0.25 |
| AT5G19800 | HYDROXYPROLINE-RICH GLYCOPROTEIN 2 (HRGP2) | Trichoblast differentiation | n.c. | 0.39 |
| AT5G20250 | RAFFINOSE SYNTHASE 6 (RS6) | Response to oxidative stress | 2.43 | n.c. |
| AT5G22410 | ROOT HAIR SPECIFIC 18 (RHS18) | Response to oxidative stress | n.c. | 0.27 |
| AT5G22555 | Unknown protein | Unknown | 0.47 | 0.32 |
| AT5G24140 | SQUALENE MONOOXYGENASE 2 (SQP2) | Sterol biosynthetic process | 0.36 | 0.20 |
| AT5G24313 | Unknown protein | Root hair cell differentiation | 0.43 | 0.38 |
| AT5G40860 | AT5G40860 | Carpel development | 0.40 | 0.26 |
| AT5G41080 | GLYCEROPHOSPHODIESTER PHOSPHODIESTERASE 2 (GDPD2) | Response to phosphate starvation | n.c. | 0.47 |
| AT5G41280 | Receptor-like protein kinase-related family protein | Unknown | n.c. | 0.35 |

| | | | | |
|---|---|---|---|---|
| AT5G42510 | Disease resistance-responsive family protein | Defense response | 0.42 | n.c. |
| AT5G49080 | EXTENSIN 11 (EXT11) | Unknown | 0.39 | 0.40 |
| AT5G49448 | CONSERVED PEPTIDE UPSTREAM OPEN READING FRAME 4 (CPUORF4) | Unknown | 2.08 | n.c. |
| AT5G49450 | BASIC LEUCINE-ZIPPER 1 (BZIP1) | Response to salt stress | 2.08 | n.c. |
| AT5G49870 | Mannose-binding lectin superfamily protein | Unknown | 0.25 | 0.17 |
| AT5G51270 | U-box domain-containing protein kinase family protein | Protein ubiquitination | 0.30 | 0.47 |
| AT5G52020 | ERF/AP2 transcription factor family protein | Regulation of transcription | n.c. | 0.37 |
| AT5G57530 | XYLOGLUCAN ENDOTRANSGLUCOSYLASE/HYDROLASE 12 (XTH12) | Trichoblast differentiation | n.c. | 0.50 |
| AT5G57540 | XYLOGLUCAN ENDOTRANSGLUCOSYLASE/HYDROLASE 13 (XTH13) | Root hair cell differentiation | n.c. | 0.39 |
| AT5G57625 | CAP (Cysteine-rich secretory proteins, Antigen 5, and Pathogenesis-related 1 protein) superfamily protein | Unknown | 0.50 | 0.42 |
| AT5G58010 | LJRHL1-LIKE 3 (LRL3) | Root hair development | 0.47 | 0.44 |
| AT5G59490 | Haloacid dehalogenase-like hydrolase (HAD) superfamily protein | Hydrolase activity | 0.37 | 0.39 |
| AT5G61260 | Calmodulin-binding protein | Unknown | n.c. | 0.49 |
| AT5G61550 | U-box domain-containing protein kinase family protein | Protein ubiquitination | n.c. | 0.45 |
| AT5G62310 | INCOMPLETE ROOT HAIR ELONGATION (IRE) | Root hair elongation | n.c. | 0.45 |
| AT5G63270 | RPM1-interacting protein 4 (RIN4) family protein | Response to nitrate | 0.48 | 0.44 |
| AT5G65160 | TETRATRICOPEPTIDE REPEAT 14 (TPR14) | Unknown | n.c. | 0.46 |
| AT5G65690 | PHOSPHOENOLPYRUVATE CARBOXYKINASE 2 (PCK2) | Gluconeogenesis | n.c. | 0.32 |
| AT5G66580 | Unknown protein | N-terminal protein myristoylation | n.c. | 0.46 |
| AT5G67400 | ROOT HAIR SPECIFIC 19 (RHS19) | Trichoblast differentiation | 0.47 | 0.43 |
| *ETC1-regulated genes* | | | | |
| AT1G15580 | INDOLE-3-ACETIC ACID INDUCIBLE 5 (IAA5) | Response to auxin | 2.46 | 0.47 |

| | | | | |
|---|---|---|---|---|
| AT2G17660 | RPM1-interacting protein 4 (RIN4) family protein | Defense response | n.c. | 0.43 |
| AT2G33460 | ROP-INTERACTIVE CRIB MOTIF-CONTAINING PROTEIN 1 (RIC1) | Pollen tube growth | n.c. | 0.38 |
| AT3G45060 | HIGH AFFINITY NITRATE TRANSPORTER 2.6 (NRT2.6) | Nitrate transport | n.c. | 0.38 |
| AT3G53150 | UDP-GLUCOSYL TRANSFERASE 73D1 (UGT73D1) | Amino acid transport | 0.49 | n.c. |
| AT3G53600 | C2H2-type zinc finger family protein | Regulation of transcription | n.c. | 0.37 |
| AT3G53820 | C2H2-type zinc finger family protein | Regulation of transcription | n.c. | 0.45 |
| AT4G22217 | Defensin-like (DEFL) family protein | Unknown | 0.46 | n.c. |
| AT5G01100 | FRIABLE 1 (FRB1) | O-fucosyltransferase family protein | n.c. | 0.42 |
| AT5G06760 | LATE EMBRYOGENESIS ABUNDANT 4-5 (LEA4-5) | Response to osmotic stress | n.c. | 0.42 |
| AT5G18910 | Protein kinase superfamily protein | Amino acid phosphorylation | 0.46 | 0.47 |
| AT5G52020 | ERF/AP2 transcription factor family protein | Regulation of transcription | n.c. | 0.40 |
| *TRY-regulated genes* | | | | |
| AT1G44090 | GIBBERELLIN 20-OXIDASE 5 (GA20OX5) | Gibberellin biosynthetic process | n.c. | 0.20 |
| AT1G70450 | Unknown protein | Enriched in root hair cells | 0.46 | n.c. |
| AT2G20670 | Domain of unknown function (DUF506) | Unknown | 2.70 | n.c. |
| AT3G53150 | UDP-GLUCOSYL TRANSFERASE 73D1 (UGT73D1) | Amino acid transport | n.c. | 0.46 |
| AT3G53820 | C2H2-type zinc finger family protein | Regulation of transcription | 0.28 | n.c. |
| AT5G01100 | FRIABLE 1 (FRB1) | O-fucosyltransferase family protein | n.c. | 0.29 |
| AT5G06760 | LATE EMBRYOGENESIS ABUNDANT 4-5 (LEA4-5) | Response to osmotic stress | 0.47 | 0.44 |
| AT5G07450 | CYCLIN P4;3 (CYCP4;3) | Regulation of cell cycle | n.c. | 0.48 |
| AT5G20250 | RAFFINOSE SYNTHASE 6 (RS6) | Response to oxidative stress | 2.27 | n.c. |
| AT5G22555 | Unknown protein | Unknown | n.c. | 0.47 |
| AT5G22920 | RING ZINC-FINGER PROTEIN 34 (RZPF34) | Regulation of stomatal opening | 2.91 | n.c. |
| AT5G38900 | Thioredoxin superfamily protein | Defense response | n.c. | 0.42 |
| AT5G49448 | CONSERVED PEPTIDE UPSTREAM OPEN READING FRAME 4 (CPUORF4) | Unknown | 2.36 | n.c. |
| AT5G49450 | BASIC LEUCINE-ZIPPER 1 (BZIP1) | Response to salt stress | 2.36 | n.c. |
| AT5G52020 | ERF/AP2 transcription factor family | Regulation of transcription | n.c. | 0.48 |

**Supplementary Table S4.** Primers used for the qRT-PCR experiments shown in Figure S2.

| Primer | Sequence |
|---|---|
| At5g20790 forward | ACCGGTCCGGTCAAAGAAA |
| At5g20790 reverse | ATCGTCAGCTTCCCCTTTGTC |
| At5g46890 forward | CTAGTTGATCTTGAAGCCGCG |
| At5g46890 reverse | GGCACATTTGAAACCAGATGG |
| *EXP7* forward | CAAGAGCACCGCTAATTTCCG |
| *EXP7* reverse | CACAACAAAAGGCCACCGAA |
| *PAP27* forward | TGCCCCATTTACCAGAACCAA |
| *PAP27* reverse | TGACTTCCTGCACCACCAACA |
| *PLP1* forward | GAACAAATCGCCCTCCAAGAA |
| *PLP1* reverse | TTACTCCGGCGATGATTCCTC |
| *PLP2* forward | AAGGCCGACCCTTATTTGCA |
| *PLP2* reverse | TTTTGGCGGCTGAGAATGG |
| *PRP3* forward | TCTTAAGGCCGTTGATGGCA |
| *PRP3* reverse | ACCTCGGTGTTGCTTTTACCG |
| *PS2* forward | GGAGCTTCATGATCAAGGCAA |
| *PS2* reverse | CATGTGCAGATTTGATGGCAG |
| *SQP2* forward | GCGACATTGCCTTTTCCAGA |
| *SQP2* reverse | GCTGCTTTTTGGCGTAAACG |
| *ZIP3* forward | GTCTTCCCTTCTTTGCGTCCA |
| *ZIP3* reverse | GCCTCAGGCAAGACATGCATA |
